# Supplementary material for: Identification of a New Antimicrobial, Desertomycin H, Utilizing a Modified Crowded Plate Technique
Source: Mar Drugs. 2021 Jul 27;19(8):424. doi: 10.3390/md19080424 (PMC8400312; doi:10.3390/md19080424)
Supplement: Supplementary file 1 [file marinedrugs-19-00424-s001.zip › marinedrugs-1309955-supplementary.pdf]

# Identification of a New Antimicrobial, Desertomycin H, Utilizing a Modified Crowded Plate Technique

Osama G. Mohamed[1,2,3], Sadaf Dorandish[4], Rebecca Lindow[4], Megan Steltz[4], Ifrah Shoukat[4], Maira Shoukat[4], Hussein Chehade[4], Sara Baghdadi[4], Madelaine McAlister-Raeburn[4,5], Asad Kamal[4,6], Dawit Abebe[4], Khaled Ali[4], Chelsey Ivy[4], Maria Antonova[4], Pamela Schultz[1,2], Michael Angell[4], Daniel Clemans[4], Timothy Friebe[7], David Sherman[2,8], Anne M. Casper[4], Paul A. Price[4*], Ashootosh Tripathi[1,2,8*]

[1] Natural Products Discovery Core, Life Sciences Institute, University of Michigan, Ann Arbor, Michigan 48109, USA

[2] Life Sciences Institute, University of Michigan, Ann Arbor, Michigan 48109, USA

[3] Pharmacognosy Department, Faculty of Pharmacy, Cairo University, Kasr el-Aini street, Cairo 11562, Egypt

[4] Biology Department, Eastern Michigan University, Ypsilanti, Michigan 48197, USA

[5] Biological Sciences, Mount Holyoke College, South Hadley, Massachusetts 01075, USA

[6] Biological Sciences, Wayne State University, Detroit, Michigan 48202, USA

[7] Chemistry Department, Eastern Michigan University, Ypsilanti, Michigan 48197, USA

[8] Department of Medicinal Chemistry, College of Pharmacy, University of Michigan, Ann Arbor, Michigan 48109, USA

KEYWORDS (antibiotic discovery, crowded plate technique, ESKAPE Pathogens, natural products, Tiny Earth®)

Corresponding authors.
*Ashootosh Tripathi, Life Sciences Institute, University of Michigan, 210 Washtenaw Avenue, Ann Arbor, Michigan 48109, USA. Email: ashtri@umich.edu; Paul Price, Biology Department, Eastern Michigan University, 441 Mark Jefferson, Ypsilanti, Michigan, 48197, USA. Email: pprice5@emich.edu

**Table of Contents:**

1. Comparison of antibiotic discovery methods ............................................................3
2. Comparison of media type for the mCPT method .......................................................3
3. Secondary Zone of Inhibition for *Streptomyces* sp. PAP62 .........................................4
4. Spectroscopic characterisation of desertomycin A (1) .................................................5
5. Spectroscopic characterisation of desertomycin H (2) ...............................................11
6. Desertomycin A and desertomycin H bioactivity .....................................................16
7. References:.................................................................................................16

**List of Figures**

Figure S1. Natural Product Extract (NPE) testing for *Streptomyces* sp. PAP62 using a drug-sensitive strain of *S. aureus*. (Left to Right) NPEs for *Streptomyces* sp. PAP62 grown in A3M (62-ASM) alone or with *C. glutamicum* (62-Corny), *M. smegmatis* (62-Myco), or *R. erythropolis* (62-Rhodo). The primary zone of inhibition on day one is denoted by the black lines. TYME plates were incubated at 37 °C and imaged at 3 days post-inoculation. ...............4
Figure S2. $^{1}$H NMR (800 MHz, methanol-$d_4$) spectrum for desertomycin A (1)......................5
Figure S3. $^{13}$C NMR (200 MHz, methanol-$d_4$) spectrum for desertomycin A (1)....................5
Figure S4. 2D NMR HSQC spectrum (methanol-$d_4$) for desertomycin A (1)..........................6
Figure S5. 2D NMR HMBC spectrum (methanol-$d_4$) for desertomycin A (1) ........................7
Figure S6. 2D NMR COSY spectrum (methanol-$d_4$) for desertomycin A (1)..........................8
Figure S7. $^{1}$H NMR (800 MHz, methanol-$d_4$) spectrum for desertomycin H (2)...................11
Figure S8. $^{13}$C NMR (200 MHz, methanol-$d_4$) spectrum for desertomycin H (2)..................11
Figure S9. 2D NMR HSQC spectrum (methanol-$d_4$) for desertomycin H (2)........................12
Figure S10. 2D NMR HMBC spectrum (methanol-$d_4$) for desertomycin H (2) .....................13
Figure S11. 2D NMR COSY spectrum (methanol-$d_4$) for desertomycin H (2)......................14
Figure S12. Desertomycin A and H bioactivity testing. (A) 30 μg of desertomycin A (A) and H (H) were tested for activity using a drug-sensitive strain of *S. aureus* and (B) a vancomycin intermediate-sensitive *S. aureus* (VISA5). DMSO (D) was used a solvent control. TYME plates were incubated at 37 °C and imaged at 24 hours post-inoculation..........................................16

**List of Tables**

Table S1 Efficiency of the modified crowded plate technique (mCPT). Comparison of isolation techniques for identifying antibiotic producers effective against *B. subtilis* using the spread-patch assay: random selection, selection of *Streptomyces* sp., Waksman's crowded plate technique (CPT), and modified crowded plate technique (mCPT)..........................................3
Table S2. Effect of media type on the mCPT. Representative experiment of the number of zones of inhibition observed over time on various types of media using D-alanine auxotrophic strains of *E. coli* or *B. subtilis* as target organisms over 2 agar plates......................................3
Table S3. 1D and 2D NMR (800 MHz, methanol-$d_4$) data for desertomycin A (1).................9
Table S4. $^{13}$C NMR (DMSO-$d_6$) data comparison of desertomycin A (1) with published data [1].............................................................................................................10
Table S5. 1D and 2D NMR (800 MHz, methanol-$d_4$) data for desertomycin H (2)...............15

## 1. Comparison of antibiotic discovery methods

**Table S1** Efficiency of the modified crowded plate technique (mCPT)**.** Comparison of isolation techniques for identifying antibiotic producers effective against *B. subtilis* using the spread-patch assay: random selection, selection of *Streptomyces* sp., Waksman's crowded plate technique (CPT), and modified crowded plate technique (mCPT).

| Screening method | Number screened | Number of producers | Efficiency (%) |
|---|---|---|---|
| Random | 210 | 13 | 6.2% |
| Streptomycete isolation | 100 | 18 | 18% |
| CPT | 250 | 62 | 25% |
| mCPT (*B. subtilis*) | 100 | 75 | 75% |

## 2. Comparison of media type for the mCPT method

**Table S2.** Effect of media type on the mCPT. Representative experiment of the number of zones of inhibition observed over time on various types of media using D-alanine auxotrophic strains of *E. coli* or *B. subtilis* as target organisms over 2 agar plates.

| | | Time | | | | |
|---|---|---|---|---|---|---|
| Medium | Target organism | Day 1 | Week1 | Week2 | Week 3 | Week 4 |
| TY | *E. coli* | 0 | 0 | 0 | 0 | 1 |
| | *B. subtilis* | 0 | 0 | 0 | 0 | 0 |
| 10% TSA | *E. coli* | 0 | 0 | 0 | 0 | 0 |
| | *B. subtilis* | 0 | 2 | 2 | 2 | 2 |
| MYM | *E. coli* | 0 | 4 | 4 | 4 | 5 |
| | *B. subtilis* | 0 | 0 | 0 | 2 | 3 |
| R2A | *E. coli* | 0 | 2 | 4 | 4 | 4 |
| | *B. subtilis* | 0 | 0 | 0 | 3 | 3 |
| TYME | *E. coli* | 0 | 8 | 9 | 12 | 13 |
| | *B. subtilis* | 0 | 3 | 4 | 8 | 8 |

## 3. Secondary Zone of Inhibition for *Streptomyces* sp. PAP62

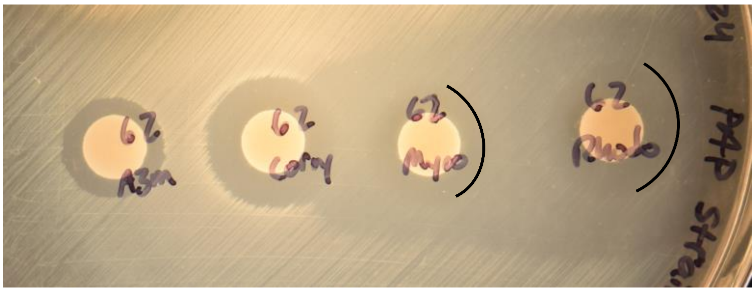

Target organism: *S. aureus*

**Figure S1.** Natural Product Extract (NPE) testing for *Streptomyces* sp. PAP62 using a drug-sensitive strain of *S. aureus*. (Left to Right) NPEs for *Streptomyces* sp. PAP62 grown in A3M (62-ASM) alone or with *C. glutamicum* (62-Corny), *M. smegmatis* (62-Myco), or *R. erythropolis* (62-Rhodo). The primary zone of inhibition on day one is denoted by the black lines. TYME plates were incubated at 37 ℃ and imaged at 3 days post-inoculation.

## 4. Spectroscopic characterisation of desertomycin A (1)

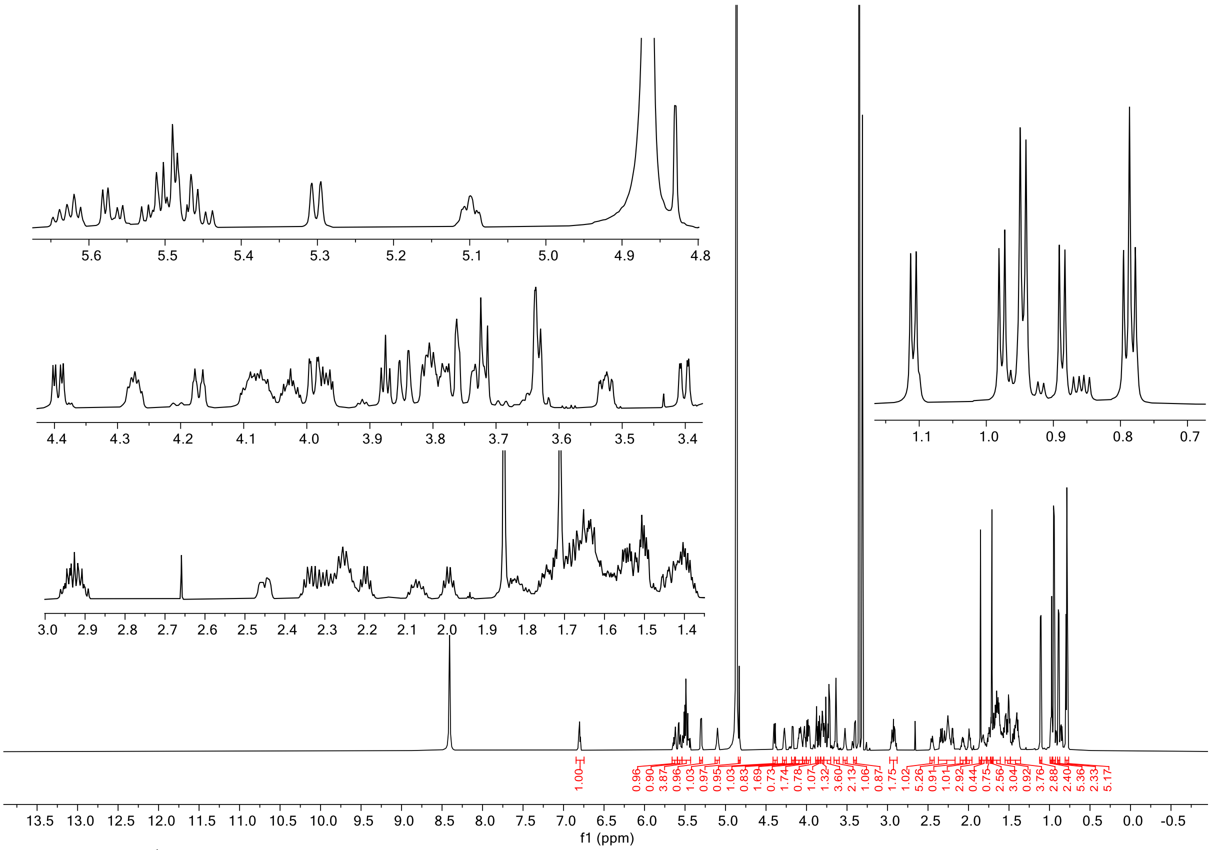


**Figure S2.** $^{1}$H NMR (800 MHz, methanol-$d_4$) spectrum for desertomycin A (**1**)

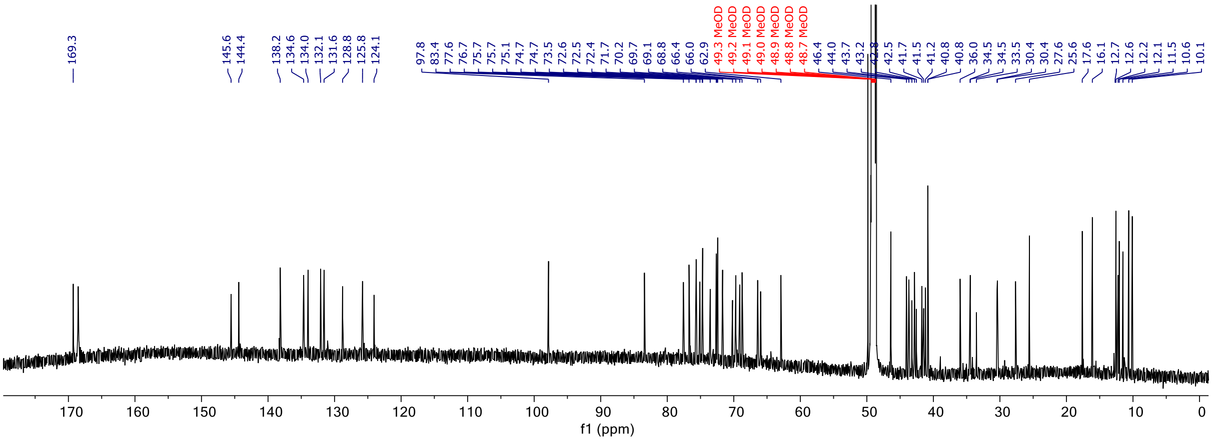


**Figure S3.** $^{13}$C NMR (200 MHz, methanol-$d_4$) spectrum for desertomycin A (**1**)

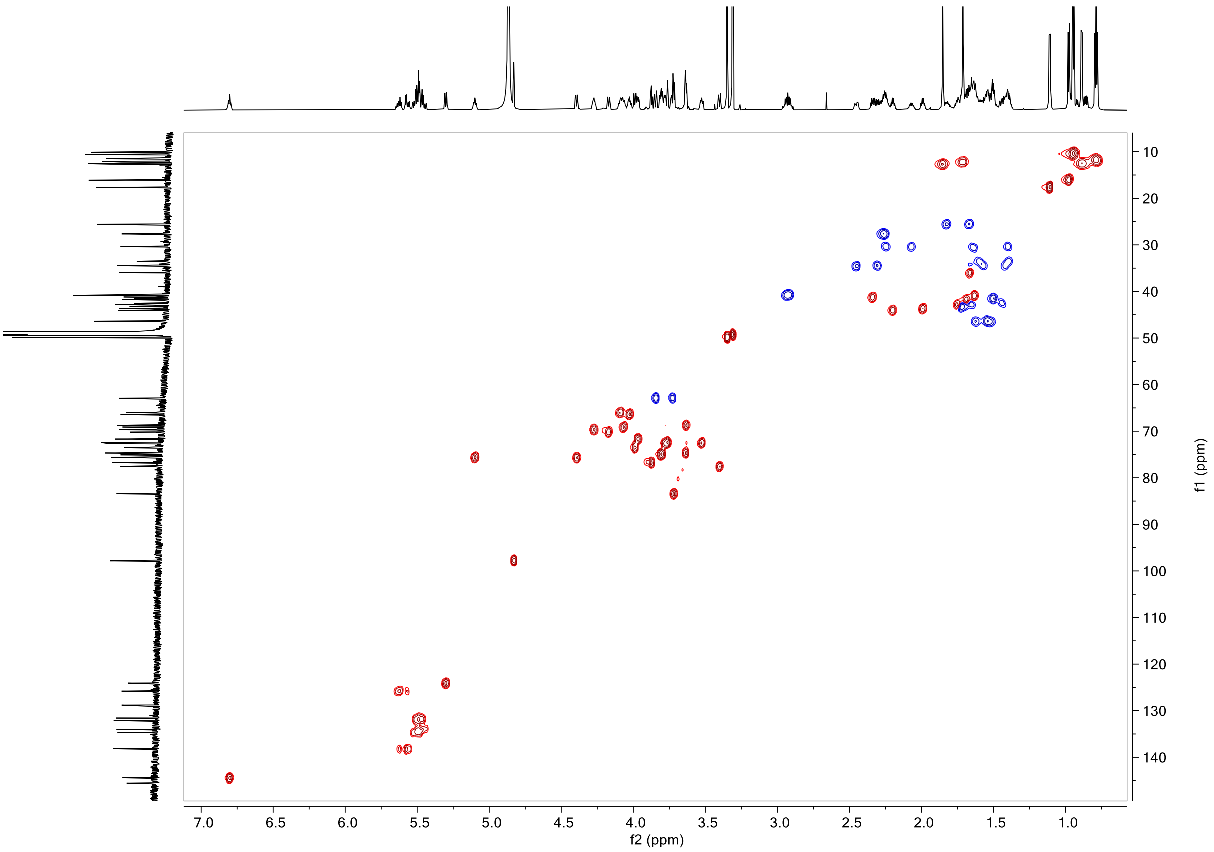


**Figure S4.** 2D NMR HSQC spectrum (methanol-$d_4$) for desertomycin A (**1**)

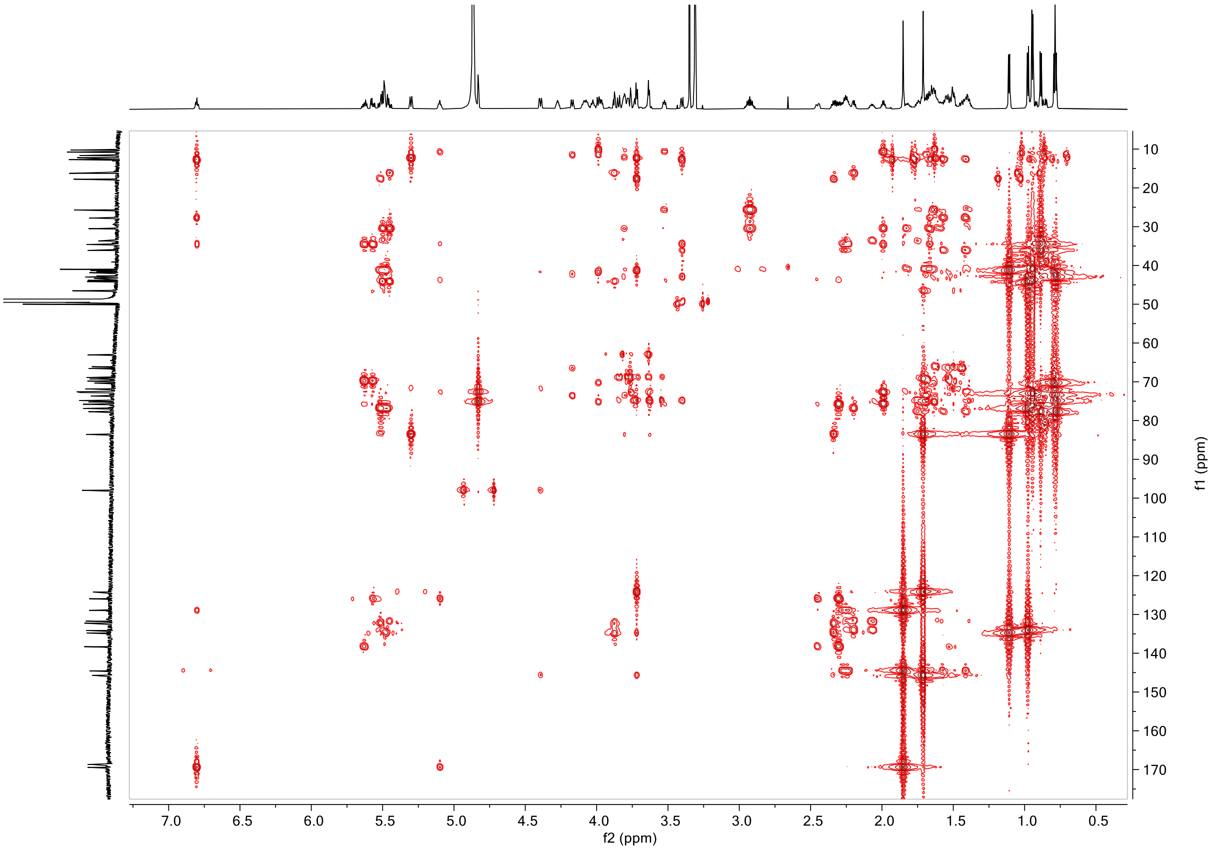


**Figure S5.** 2D NMR HMBC spectrum (methanol-$d_4$) for desertomycin A (**1**)

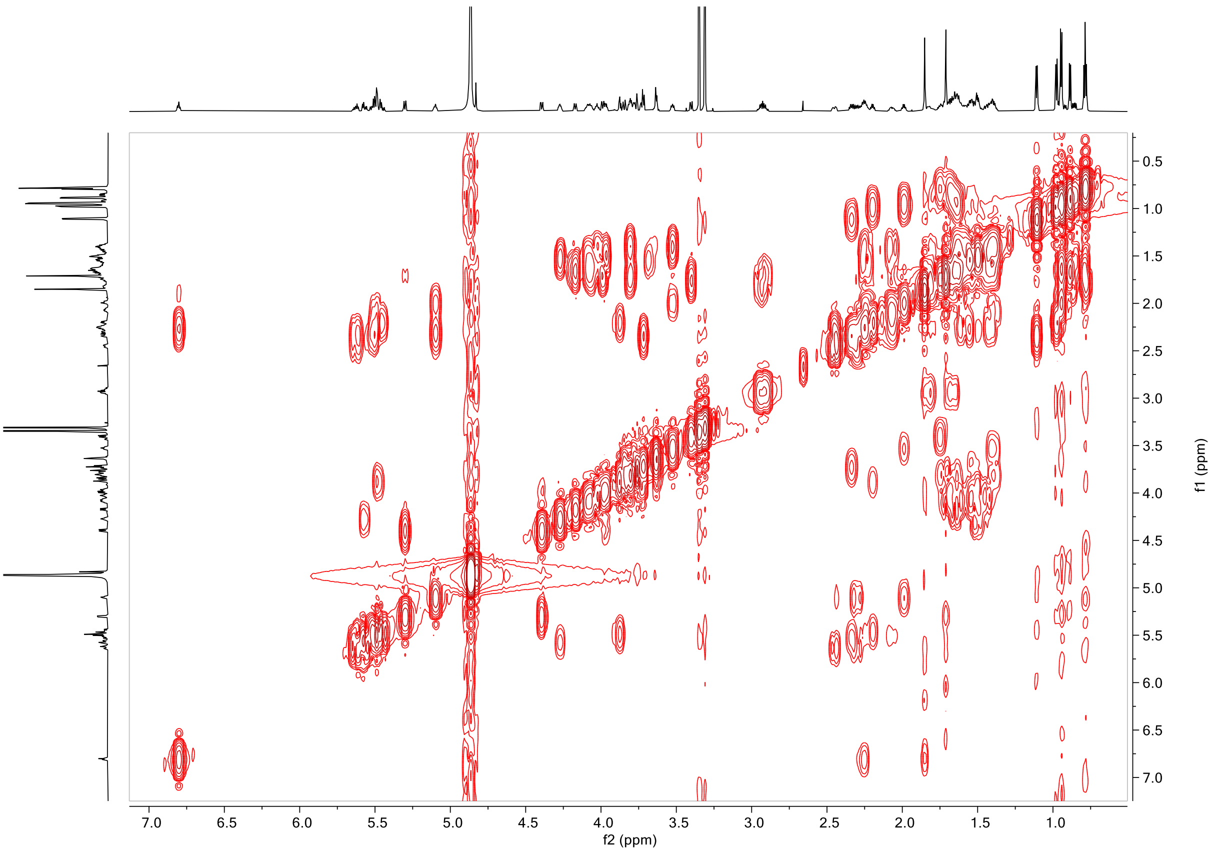


**Figure S6.** 2D NMR COSY spectrum (methanol-$d_4$) for desertomycin A (**1**)

**Table S3.** 1D and 2D NMR (800 MHz, methanol-$d_4$) data for desertomycin A (**1**)

| Pos. | $\delta_H$, m (*J* Hz) | $\delta_C$ | *COSY* | *HMBC* | Pos. | $\delta_H$, m (*J* Hz) | $\delta_C$ | *COSY* | *HMBC* |
|---|---|---|---|---|---|---|---|---|---|
| 1 | --- | 169.3 | | | 33 | 4.17, td (*10.0*, *2.3*) | 70.2 | 32, 34 | |
| 2 | --- | 128.8 | | | 34 | 1.69[b] | 43.2 | 33 | |
| 3 | 6.80, m | 144.4 | 4 | 1, 2, 4, 5 | 35 | 4.02, m | 66.5 | 34, 36 | |
| 4 | 2.26[a], m | 27.6 | 3 | 2, 3, 5, 6 | 36 | 1.53[g] | 46.4 | 35, 37 | |
| 5 | a 1.42, m | 34.5 | 4, 6 | 3, 4, 6, 7 | 37 | 4.27, m | 69.7 | 36, 37 | 34, 35, 38, 39 |
| | b 1.57, m | | | | 38 | 5.58, dd (*15.4*, *5.5*) | 138.2 | 37, 39 | 36, 37, 39, 40 |
| 6 | 1.67[b], m | 36.0 | 48 | | 39 | 5.62, dd (*15.4*, *6.7*, *0.9*) | 125.8 | 38, 40a | 37, 38, 40, 41 |
| 7 | 3.40, dd (*9.6*, *1.7*) | 77.5 | 8 | | 40 | a 2.30, dd (*13.4*, *6.7*) | 34.5 | 39, 40b | |
| 8 | 1.75, m | 42.8 | 49 | | | b 2.45, dd (*13.4*, *5.3*) | | 39, 40b, 41 | |
| 9 | 3.80[c] | 74.7[k] | 10a, 10b | | 41 | 5.10, m | 75.6[l] | 40b, 42 | |
| 10 | a 1.40[d] | | 9 | | 42 | 1.99, q (*6.9*) | 43.7 | 41, 43, 55 | |
| | b 1.60[e] | | 9 | | 43 | 3.52, ddd (*9.8*, *5.6*, *1.9*) | 72.6 | 42, 44a, 44b | |
| 11 | a 2.07, m | 30.40 | 10a, 11b | | 44 | a 1.40[d] | 30.44 | 43, 44b, 45 | |
| | b 2.24[a] | | 10a, 10b, 11a | | | b 1.63[e] | | 43, 44a, 45 | |
| 12 | 5.49[f] | 131.6 | 11a, 11b, 13 | 10, 11, 13, 14 | 45 | a 1.67[b] | 25.6 | 44a, 44b, 46 | |
| 13 | 5.45[f] | 134.0 | 12, 14 | 11, 12, 14, 15 | | b 1.82, m | | | |
| 14 | 2.19, q (*6.7*) | 44.1 | | | 46 | 2.93, m | 40.8 | 45 | 44, 45 |
| 15 | 3.87, dd (*6.7*, *5.5*) | 76.7 | 14 | | 47 | 1.85, s | 12.7 | 3 | 1, 2, 3 |
| 16 | 5.49[f] | 132.1 | 15, 17 | 14, 15, 17, 18 | 48 | 0.88, d (*6.8*) | 12.6 | 6 | 5, 6, 7 |
| 17 | 5.50[f] | 134.7 | 16, 18 | 14, 15, 18, 19, 51 | 49 | 0.78, d (*6.9*) | 12.0 | 8 | 7, 8, 9 |
| 18 | 2.34, m | 41.2 | 19, 51 | | 50 | 0.97, d (*6.8*) | 16.1 | 14 | 13, 14, 15 |
| 19 | 3.72, d (*8.5*) | 83.4 | 18 | | 51 | 1.11, d (*6.9*) | 17.6 | 18 | 17, 18, 19 |
| 20 | --- | 145.5 | | | 52 | 1.71, s | 12.2 | | 19, 20, 21 |
| 21 | 5.30, d (*9.6*) | 124.1 | 22 | | 53 | 0.94[h], d (*6.9*) | 10.1 | 30 | 29, 30, 31 |
| 22 | 4.39, dd (*9.6*, *3.5*) | 75.6[l] | 21 | | 54 | 0.79, d (*7.0*) | 11.5 | 32 | 31, 32, 33 |
| 23 | 3.96, dd (*8.3*, *3.5*) | 71.7 | 24 | | 55 | 0.94[h], d (*6.9*) | 10.6 | 42 | 41, 42, 43 |
| 24 | 1.49, m | 41.5 | 23 | | 1’ | 4.83, d (*1.5*) | 97.8 | | 22, 2’, 3’, 5’ |
| 25 | 4.08, m | 66.0 | 26a, 26b | | 2’ | 3.76[i] | 72.4* | | |
| 26 | a 1.53[g] | 46.4 | 25 | | 3’ | 3.76[i] | 72.5* | | |
| | b 1.62[e] | | 25 | | 4’ | 3.63[j] | 68.7 | | |
| 27 | 4.06, m | 69.1 | 26, 28 | | 5’ | 3.63[j] | 74.7[k] | | |
| 28 | 1.44, m | 42.5 | 27 | | 6’ | a 3.72, dd (*11.5*, *3.6*) | 62.9 | | |
| 29 | 3.80[c] | 75.1 | 28, 30 | | | b 3.84, dd (*11.5*, *1.3*) | | | |
| 30 | 1.63[e] | 40.8 | 31, 53 | | | | | | |
| 31 | 3.98, dd (*9.5*, *1.7*) | 73.5 | 30 | | | | | | |
| 32 | 1.69[b] | 41.75 | 54 | | | | | | |

[a-l] overlapping signals within the same superscript letter, * interchangeable assignments

**Table S4.** $^{13}$C NMR (DMSO-$d_6$) data comparison of desertomycin A (**1**) with published data [1]

| Pos. | $\delta_c$, experimental | $\delta_C$ literature | Pos. | $\delta_c$, experimental | $\delta_C$ literature |
|---|---|---|---|---|---|
| 1 | 166.5 | 166.5 | 32 | 40.5 | 40.3 |
| 2 | 127.1 | 127.1 | 33 | 66.7 | 66.7 |
| 3 | 142.1 | 142.1 | 34 | 42.2 | 41.9 |
| 4 | 26.1 | 26.1 | 35 | 63.7 | 63.7 |
| 5 | 33.0 | 32.5 | 36 | 46.0 | 45.9 |
| 6 | 34.4 | 34.4 | 37 | 66.7 | 66.8 |
| 7 | 74.6 | 74.9 | 38 | 138.0 | 137.6 |
| 8 | 41.4 | 41.4 | 39 | 122.8 | 122.9 |
| 9 | 72.0 | 72.1 | 40 | 32.1 | 32.1 |
| 10 | 32.5 | 32.4 | 41 | 73.6 | 73.5 |
| 11 | 29.1 | 29.0 | 42 | 41.8 | 41.7 |
| 12 | 129.6 | 129.5 | 43 | 70.2 | 69.4 |
| 13 | 132.6 | 132.6 | 44 | 30.0 | 29.9 |
| 14 | 42.2 | 42.1 | 45 | 25.0* | 24.0 |
| 15 | 74.4 | 74.4 | 46 | 39.2 | 39 |
| 16 | 131.1 | 130.9 | 47 | 12.3 | 12.0 |
| 17 | 132.7 | 132.7 | 48 | 12.3 | 12.2 |
| 18 | 39.2 | 39.2 | 49 | 11.2 | 11.6 |
| 19 | 80.9 | 80.9 | 50 | 15.3 | 15.3 |
| 20 | 143.3 | 143.3 | 51 | 16.8 | 16.8 |
| 21 | 122.3 | 122.3 | 52 | 11.6 | 11.6 |
| 22 | 73.6 | 73.6 | 53 | 9.7 | 9.7 |
| 23 | 69.4 | 69.5 | 54 | 10.5 | 11.2 |
| 24 | 40.9 | 40.6 | 55 | 10.4 | 11.2 |
| 25 | 63.3 | 63.3 | 1’ | 95.9 | 95.9 |
| 26 | 45.8 | 45.7 | 2’ | 70.5 | 70.1 |
| 27 | 66.5 | 66.4 | 3’ | 71.0 | 70.6 |
| 28 | 42.4 | 42.1 | 4’ | 67.2 | 67.2 |
| 29 | 72.8 | 72.8 | 5’ | 73.5 | 73.5 |
| 30 | 39.3 | 39.3 | 6’ | 61.3 | 61.3 |
| 31 | 70.6 | 70.6 | | | |

*signal determined by HSQC

## 5. Spectroscopic characterisation of desertomycin H (2)

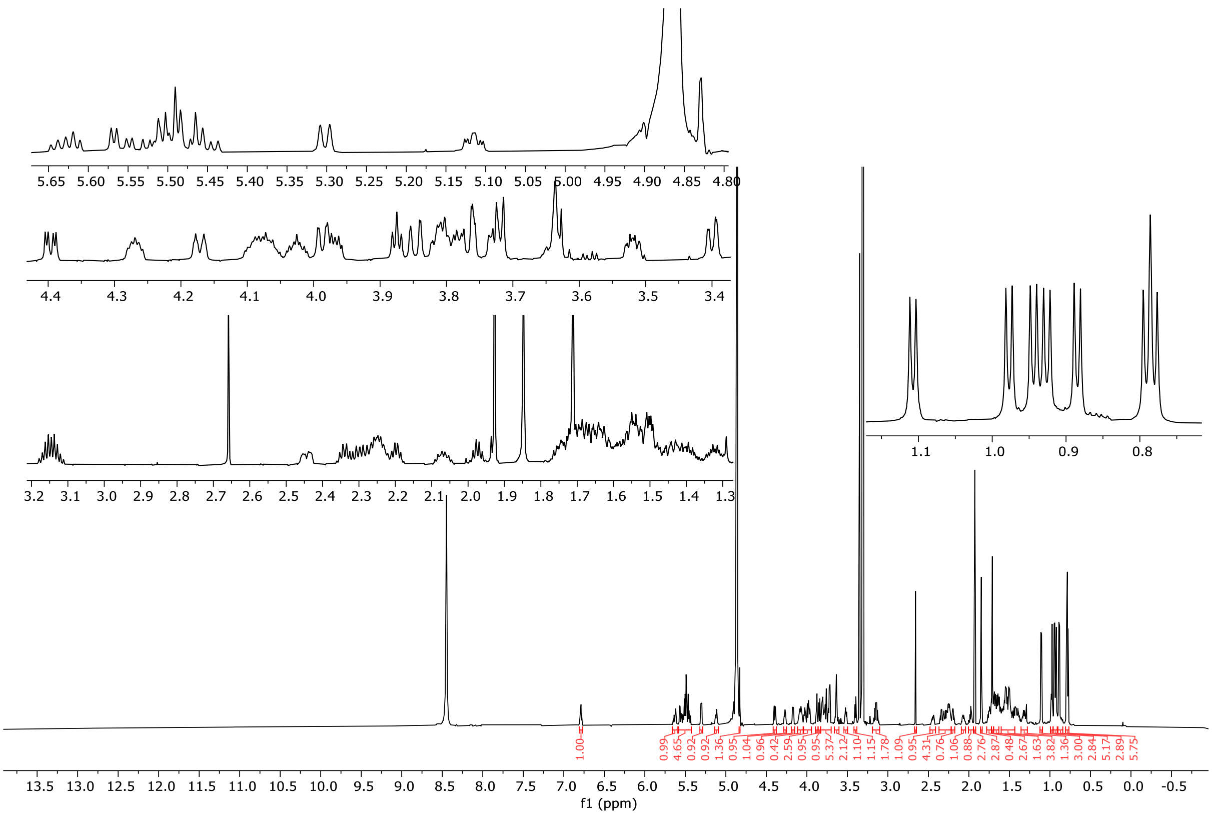

**Figure S7.** $^{1}H$ NMR (800 MHz, methanol-*d*$_4$) spectrum for desertomycin H (**2**)

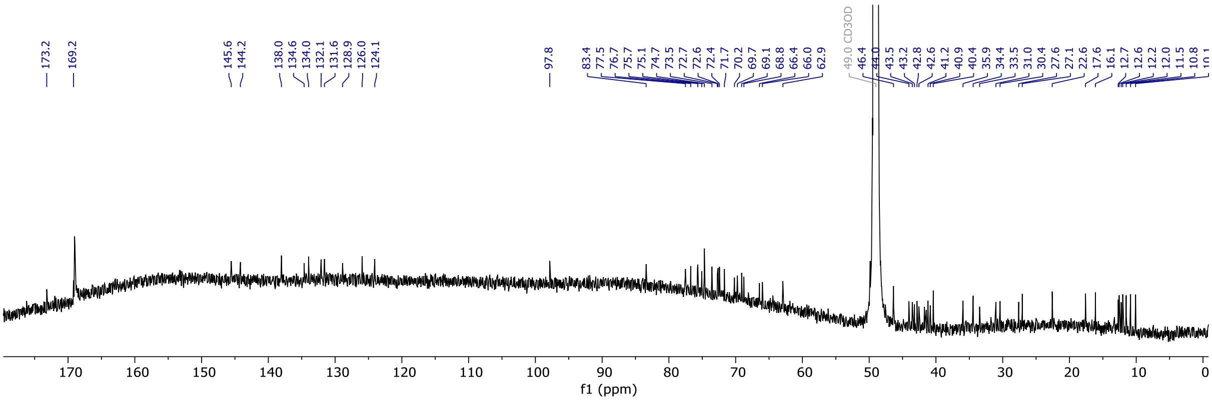

**Figure S8.** $^{13}C$ NMR (200 MHz, methanol-*d*$_4$) spectrum for desertomycin H (**2**)

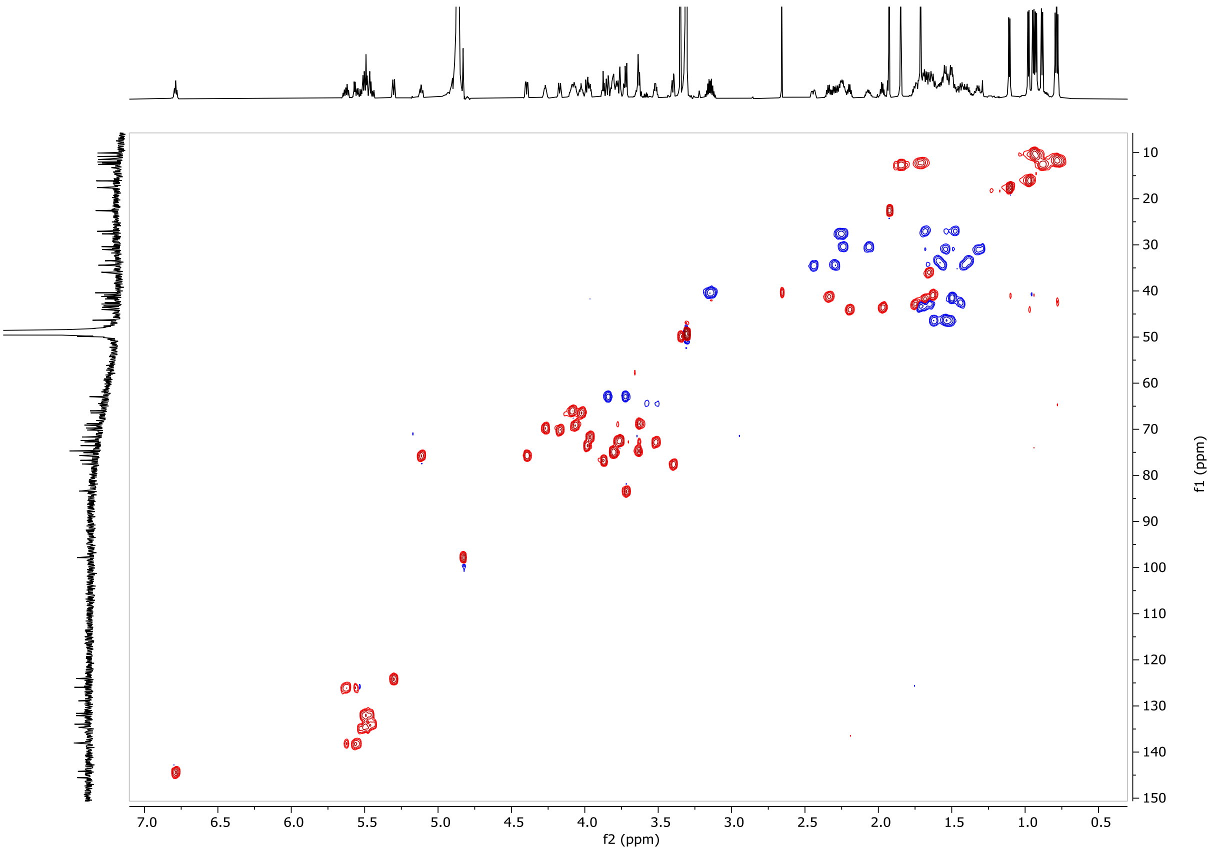


**Figure S9.** 2D NMR HSQC spectrum (methanol-$d_4$) for desertomycin H (**2**)

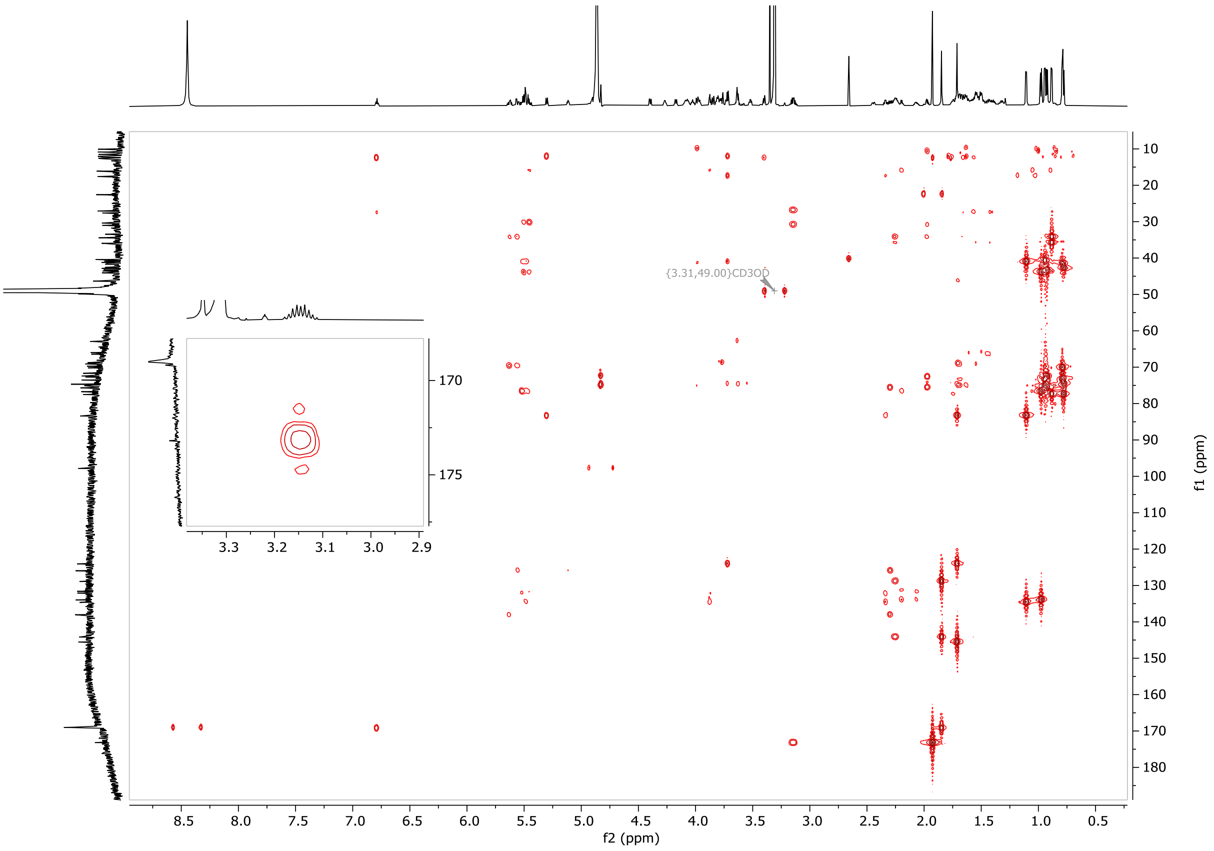


**Figure S10.** 2D NMR HMBC spectrum (methanol-$d_4$) for desertomycin H (**2**)

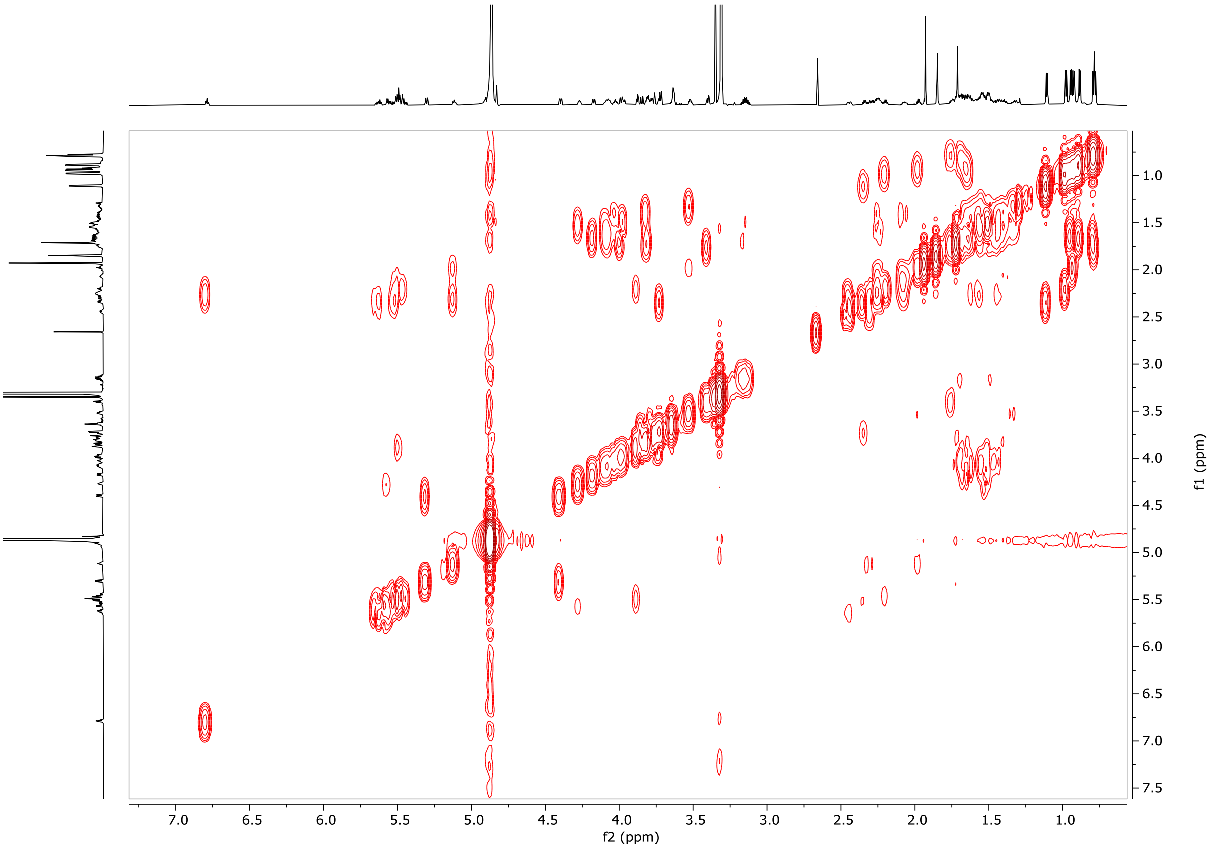


**Figure S11.** 2D NMR COSY spectrum (methanol-$d_4$) for desertomycin H (**2**)

**Table S5.** 1D and 2D NMR (800 MHz, methanol-$d_4$) data for desertomycin H (**2**)

| Pos. | $\delta_H$, m (*J* Hz) | $\delta_C$ | *COSY* | *HMBC* | Pos. | $\delta_H$, m (*J* Hz) | $\delta_C$ | *COSY* | *HMBC* |
|---|---|---|---|---|---|---|---|---|---|
| 1 | --- | 169.2 | | | 33 | 4.17, td (*10.0*, *2.0*) | 70.2 | 32, 34 | |
| 2 | --- | 128.9 | | | 34 | 1.70[b] | 43.2 | 33 | |
| 3 | 6.79, m | 144.2 | 4 | 1, 2, 4, 5 | 35 | 4.01, m | 66.4 | 34, 36 | |
| 4 | 2.25[a], m | 27.6 | 3 | 2, 3, 5, 6 | 36 | 1.52[f] | 46.3[l] | 35, 37 | |
| 5 | a 1.41, m | 34.4[i] | 4, 6 | 3, 4, 6, 7 | 37 | 4.27, m | 69.7 | 36, 37 | 34, 35, 38, 39 |
| | b 1.56, m | | | | 38 | 5.56, dd (*15.5*, *5.7*) | 138.0 | 37, 39 | 36, 37, 39, 40 |
| 6 | 1.65[b], m | 35.9 | 48 | | 39 | 5.62, dd (*15.0*, *7.2*) | 125.9 | 38, 40a | 37, 38, 40, 41 |
| 7 | 3.39, dd ( | 77.5 | 8 | | 40 | a 2.29, dd (*13.5*, *7.2*) | 34.4[i] | 39, 40b | |
| 8 | 1.74, m | 42.8 | 49 | | | b 2.44, dd (*13.5*, *5.1*) | | 39, 40b, 41 | |
| 9 | 3.80[c] | 74.7[j] | 10a, 10b | | 41 | 5.11, m | 75.7[k] | 40b, 42 | |
| 10 | a 1.38 | 33.5 | 9 | | 42 | 1.97, q (*6.8*) | 43.5 | 41, 43, 55 | |
| | b 1.59[d] | | 9 | | 43 | 3.50, ddd (*8.4*, *5.5*, *1.8*) | 72.7 | 42, 44a, 44b | |
| 11 | a 2.07, m | 30.4 | 10a, 11b | | 44 | a 1.31 | 31.0 | 43, 44b, 45 | |
| | b 2.24[a] | | 10a, 10b, 11a | | | b 1.54[f] | | 43, 44a, 45 | |
| 12 | 5.49[e] | 131.6 | 11a, 11b, 13 | 10, 11, 13, 14 | 45 | a 1.48[f] | 27.1 | 44a, 44b, 46 | |
| 13 | 5.46[e] | 134.0 | 12, 14 | 11, 12, 14, 15 | | b 1.68[b] | | | |
| 14 | 2.19, q (*6.6*) | 44.0 | | | 46 | 3.15, m | 40.4 | 45 | 44, 45, 46-NHCOCH3 |
| 15 | 3.87, dd (6.6, 5.3) | 76.7 | 14 | | 46-NHCOCH3 | --- | 173.2 | | |
| 16 | 5.46[e] | 132.1 | 15, 17 | 14, 15, 17, 18 | 46-NHCOCH3 | 1.92, s | 22.6 | | 46, 46-NHCOCH |
| 17 | 5.49[e] | 134.6 | 16, 18 | 14, 15, 18, 19, 51 | 47 | 1.85, s | 12.7 | 3 | 1, 2, 3 |
| 18 | 2.33, m | 41.2 | 19, 51 | | 48 | 0.88, d (*6.8*) | 12.5 | 6 | 5, 6, 7 |
| 19 | 3.71, d | 83.4 | 18 | | 49 | 0.78, d (*7.5*) | 12.0 | 8 | 7, 8, 9 |
| 20 | --- | 145.6 | | | 50 | 0.98, d (*6.9*) | 16.1 | 14 | 13, 14, 15 |
| 21 | 5.30, d (*9.3*) | 124.0 | 22 | | 51 | 1.11, d (*6.6*) | 17.6 | 18 | 17, 18, 19 |
| 22 | 4.39, dd (*9.6*, *3.6*) | 75.7[k] | 21 | | 52 | 1.71, s | 12.2 | | 19, 20, 21 |
| 23 | 3.96, dd (*8.0*, *3.4*) | 71.7 | 24 | | 53 | 0.94, d (*6.9*) | 10.1 | 30 | 29, 30, 31 |
| 24 | 1.49, m | 41.46 | 23 | | 54 | 0.79, d (*7.5*) | 11.5 | 32 | 31, 32, 33 |
| 25 | 4.08, m | 66.0 | 26a, 26b | | 55 | 0.92, d (*6.9*) | 10.8 | 42 | 41, 42, 43 |
| 26 | a 1.53[f] | 46.3[l] | 25 | | 1’ | 4.83, d (*1.3*) | 97.8 | | 22, 2’, 3’, 5’ |
| | b 1.62[d] | | 25 | | 2’ | 3.77[g] | 72.4* | | |
| 27 | 4.07, m | 69.1 | 26, 28 | | 3’ | 3.75[g] | 72.6* | | |
| 28 | 1.44, m | 42.55 | 27 | | 4’ | 3.63[h] | 68.7 | | |
| 29 | 3.80[c] | 75.1 | 28, 30 | | 5’ | 3.63[h] | 74.7[j] | | |
| 30 | | | 31, 53 | | 6’ | a 3.72 | 62.9 | | |
| 31 | 3.98, dd (*9.9*, *1.7*) | 73.5 | 30 | | | b 3.84 | | | |
| 32 | 1.68[b] | 41.7 | 54 | | | | | | |

[a-l] overlapping signals within the same superscript letter, * interchangeable assignments

## 6. Desertomycin A and desertomycin H bioactivity

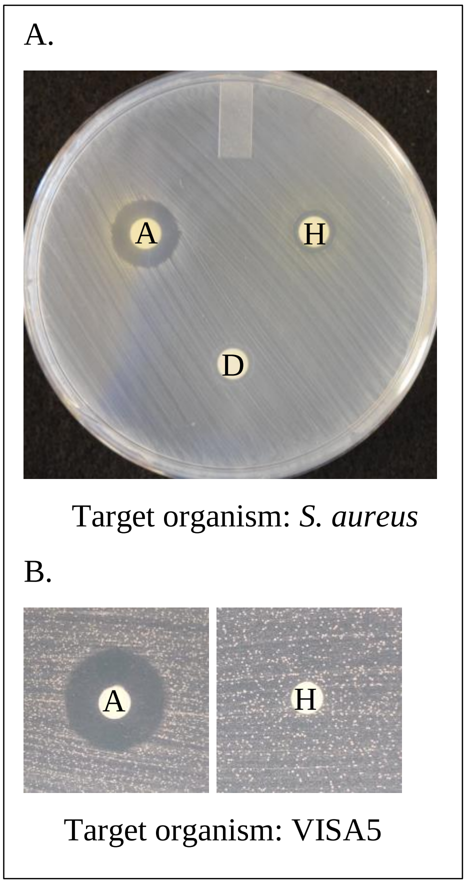


**Figure S12.** Desertomycin A and H bioactivity testing. (A) 30 μg of desertomycin A (A) and H (H) were tested for activity using a drug-sensitive strain of *S. aureus* and (B) a vancomycin intermediate-sensitive *S. aureus* (VISA5). DMSO (D) was used a solvent control. TYME plates were incubated at 37 ℃ and imaged at 24 hours post-inoculation.

## 7. References:

1. Ivanova, V. New Macrolactone of the Desertomycin Family from Sttreptomyces Spectabilis. *Preparative biochemistry & biotechnology* **1997**, *27*, 19-38.
